# Talking about Risk, UncertaintieS of Testing IN Genetics (TRUSTING): development and evaluation of an educational programme for healthcare professionals about *BRCA1 & BRCA2* testing

**DOI:** 10.1038/s41416-022-01871-x

**Published:** 2022-06-17

**Authors:** Lesley Fallowfield, Ivonne Solis-Trapala, Rachel Starkings, Shirley May, Lucy Matthews, Diana Eccles, D. Gareth Evans, Clare Turnbull, Gillian Crawford, Valerie Jenkins

**Affiliations:** 1 Sussex Health Outcomes Research & Education in Cancer (SHORE-C), Brighton & Sussex Medical School, University of Sussex, Falmer, UK; 2 Faculty of Medicine and Health Sciences, Keele University, Keele, UK; 3 School of Cancer Sciences, Faculty of Medicine, University of Southampton, Southampton, UK; 4 Division of Evolution Infection and Genomic Sciences, University of Manchester, Manchester Academic Health Science Centre, Manchester, UK; 5 Division of Genetics and Epidemiology, Institute of Cancer Research, London, UK; 6 Wessex Clinical Genetics Service, University Hospital Southampton NHS Foundation Trust, Southampton, UK

**Keywords:** Breast cancer, Cancer

## Abstract

**Background:**

Mainstreaming of germline testing demands that all healthcare professionals have good communication skills, but few have genetic testing and counselling experience. We developed and evaluated educational workshops—Talking about Risk & UncertaintieS of Testing IN Genetics (TRUSTING). Contents included: presentations and exercises, an interview with a geneticist about *BRCA* testing, screening and prevention implications, filmed interactions between surgeons, a genetic counsellor and geneticists with a fictitious family (proband had a *BRCA2* pathogenic variant with triple-negative breast cancer, her older sister—*BRCA2* heterozygous, and cousin—negative for *BRCA2* variant).

**Methods:**

Twenty-one surgeons, 5 oncologists, 18 nurses and 9 genetic counsellors participated. Knowledge (18 item MCQ), communication skills (responses to 6 questions from proband and relatives) and self-confidence (discussing 9 genetic testing issues) were assessed pre- and post workshop.

**Results:**

Knowledge scores improved significantly post workshop (mean change = 7.06; 95% confidence interval (CI) 6.37–7.74; *P* < 0.001), as did communication (mean change = 5.38; 95% CI 4.37–6.38; *P* < 0.001) and self-confidence (*P* < 0.001).

**Discussion:**

Healthcare professionals’ knowledge and self-confidence when discussing the risks and uncertainties in genetics are often poor. TRUSTING workshops significantly enhanced attendees’ navigation of communication difficulties encountered and will be rolled out more widely.

## Introduction

There are increasing calls for genetic testing of all patients with breast cancer [1]. Such testing can help determine appropriate treatment, surveillance or prevention and options for individuals with breast cancer and any family members shown to carry pathogenic gene variants (faults). Many family cancer genetics services cannot easily cope with the burgeoning demand and referrals often experience long waiting times for counselling and testing. To circumvent this, mainstream clinical genetic testing programmes have been developed and initial evaluations of these are broadly positive [2]. However, there are some difficulties, cancer clinicians are not necessarily experienced in genetics or in dealing with some of the potentially challenging and complex conversations that may arise about risk and the uncertainties of genetic testing. A systematic review of the barriers and facilitators associated with mainstreaming also concluded that many nurses and physicians had limited knowledge and skills so felt largely unprepared to integrate genetic information into routine care [3]. Another UK study revealed that breast surgeons in particular were less keen on the implementation of mainstreaming, citing a lack of time and expertise in counselling about genetic testing [4].

All Health Care Professionals (HCPs) working within cancer settings may be faced with issues such as: (1) discussing genetic testing for a germline cancer susceptibility gene; (2) dealing with the consequences of high-risk genetic diagnoses such as *BRCA1/2* or Lynch Syndrome; (3) helping patients to contextualise such information correctly; (4) assisting patients’ decision-making about further risk-reducing treatment options; and (5) facilitating the sharing of information with family members. Many of these conversations present different challenges that few HCPs have received sufficient training to deal with adequately.

Disclosing the identification of a high-risk genetic susceptibility to breast cancer at the same time as a patient is dealing with their current cancer treatment requires particularly sensitive communication. The implications of any genetic variations have to be conveyed and understood by the patient in a context relevant to their particular cancer diagnosis and treatment. For example, the POSH study showed that young-onset breast cancer patients with symptomatic breast cancer have similar overall medium-term survival comparing *BRCA1/2* carriers to non-carriers [5]. One implication of this being that for some patients it may be appropriate to delay decisions regarding risk-reducing surgery. With modern MRI-based annual screening, patients may prefer to delay decisions about additional surgery until psychologically and physically recovered from their initial treatment. Likewise, risk-reducing bilateral salpingo-oophorectomy for younger women with a *BRCA1/BRCA2* pathogenic variant might be scheduled in the future following more thought and discussion about the patient’s desire for pregnancy and the consequences of early oestrogen deprivation.

One beneficial outcome of a positive genetic diagnosis (for a pathogenic variant) is that unaffected relatives who carry the same high-risk gene variant can access effective enhanced surveillance or prevention opportunities. A survey of 11,766 women with breast cancer showed the importance of good communication. Respondents reported that mere acquisition of risk information via genetic counselling contributed to a basic level of understanding but that effective communication about risk and uncertainty with salient others, including trusted oncologists and healthcare providers, were an 'essential component' for women to feel that they had sufficient information to discuss breast cancer risk with their children [6].

There are many considerations that are not merely limited to the best way in which to present risk information including the implications of increased risk awareness for an individual and their family that might result in potential harms as well as benefits. For higher-risk individuals, concerns include the inevitable anxiety, a possibility of overtreatment, the complications of risk-reducing surgery (RRS), and worries about insurance, lifestyle choices, having children, to name but a few. There is also disquiet about providing false reassurance to those shown to be at lower risk.

We wished to produce evidence-based materials that could later be made available to appropriate trainers and facilitators thus maximising a cascade of educational opportunities for junior and senior HCPs. Using similar methods to those employed in previous successful educational projects such as (TARGET) [7], looking at communication about gene expression profile testing, we developed the **T**alking about **R**isk, **U**ncertaintie**S** of **T**esting **IN G**enetics (**TRUSTING**) programme). Central to the success of this educational model are the ideas of Knowles, Friere, Engel and Lipkin [8–11] who postulated that adult learners benefit most from courses that integrate exercises and activities designed to create simultaneous rather than sequential communication skills development (CSD), knowledge acquisition (KA) and personal awareness (PA). If learners know what to do but not how to do it then clumsy, inflexible application is likely and if they know what and how but do not believe in it or lack self-confidence they will not use in practice. To enhance these learning objectives TRUSTING workshops incorporated: (1) didactic components namely a lecture about the psychology of risk and uncertainty and a filmed interview with a geneticist about pathogenic variants and implications for the proband and her relatives (KA), (2) exercises exploring participants’ own numeracy skills, their tolerance of uncertainty and self-confidence when discussing different aspects of *BRCA1* and *BRCA2* testing and results (PA) and (3) facilitator-led group discussion of 6 filmed scenarios with members of a fictitious family in which the proband had triple-negative breast cancer (TNBC) and a *BRCA2* pathogenic variant (CSD). Thus, all elements of the programme were designed to improve (HCPs) communication skills, knowledge and self-confidence when talking to individuals about genetic risk, testing, results and implications. We report here an evaluation of the educational programme.

NB: The correct terminology for a change in the genetic code of a given cancer susceptibility gene that leads to a moderate or high increase in lifetime cancer is a pathogenic variant. This is not a term that patients or clinicians typically use in discussions about genetic testing or results so during the development of the materials, various more commonly used terms were used to describe a pathogenic variant in BRCA1 or BRCA2. For the purposes of this manuscript, this informal terminology is clarified with the technically accurate “pathogenic variant” in parentheses.

## Methods

### Workshop development and contents

There were four phases to the study:

#### Phase 1

During Phase 1, we conducted a systematic review of all relevant communication skills training programmes dealing with the genetic testing of high-risk breast cancer patients. We determined their content, methods of delivery, how outcomes had been measured and how successful programmes had been [12].

#### Phase 2

In Phase 2, we held three focus groups with 32 *BRCA1/2* pathogenic variant carriers in Southampton, Brighton and Manchester who described their own communication experiences including recall and understanding of risk, their attitudes to risk-reducing options and problems telling other family members that they too may need testing. Subsequently, we conducted more in-depth interviews with 11 of these women, all were *BRCA1 or BRCA2* pathogenic variant heterozygotes, and 5 of whom had or were currently being treated for breast cancer [13].

Having established the views of affected families we then had many informal discussions with surgeons, oncologists, specialist nurses, genetic counsellors and geneticists about their own acknowledged communication difficulties, potential training needs and perceptions of other colleagues’ communication difficulties. We also investigated further the primary characteristics of patients and problematic situations that hampered communication about genetics at an educational communication skills day held for a multidisciplinary group.

#### Phase 3

**Fig. 1:**
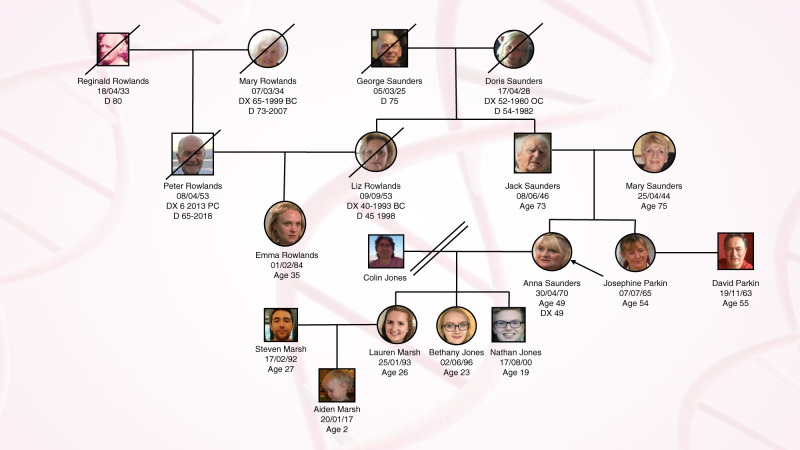
Fictious family tree developed for the TRUSTING educational materials. Shows cancer diagnoses and deaths of Anna Saunders’ (the proband) family members.

Using the information gained from Phase 2, we generated a fictitious family (see Fig. 1) created characters and developed scenarios to film in Phase 3. Our proband was aged 49, had TNBC, was a *BRCA2* carrier and had many anxieties about treatment options and implications for her family. Her sister aged 54 was also a *BRCA2* carrier with concerns about the preventative, risk-reducing measures available to her including continuing to take HRT. Finally, we introduced a younger anxious cousin in whom no *BRCA2* pathogenic variant was found but was insistent on having bilateral risk-reducing surgery. Patient simulators (actors) experienced in improvisation were filmed in six unscripted consultations with two surgeons, two geneticists (DE, CT) and a genetic counsellor (GC). This methodology had proved successful in our previous educational initiatives improving communication about risk when discussing gene expression profiling test results [7].

We also filmed an interview with a geneticist (GE) about the underlying science, implications for testing and recommendations for the fictitious family and those in general who had gene alterations, areas with which all HCPs advising patients should be familiar.

The TRUSTING workshops were designed to be conducted face-to-face in small groups; they were primarily interactive with some didactic presentations, relevant group exercises and facilitator-led discussion of the filmed scenarios. Topics covered include risk awareness of cancer amongst the general population, health literacy and numeracy (of patients and clinicians), ethical implications surrounding genetic testing, and the latest research-based evidence about ways to present complex information about risk.

#### Phase 4

Materials were reviewed by all authors and different parts piloted amongst 4 genetic counsellors, 12 surgeons, 3 specialist breast care/consultant nurses, 2 oncologists and 4 psychologists and the final programme was refined in Phase 4.

We then conducted an evaluation of the efficacy and acceptability of 8-h TRUSTING workshops with multidisciplinary HCPs.

### Participants

An advertising flyer with dates and information about TRUSTING residential workshops was circulated to potential participants via Breast Cancer meetings, geneticists, Tweets, word of mouth, the Association of Breast Cancer Surgeons, Association of Genetic Nurses and Counsellors and the SHORE-C website newsfeed (https://shore-c.sussex.ac.uk).

We aimed to recruit at least 50 participants to provide narrow 95% confidence intervals of mean post-workshop changes in scores of HCPs’ knowledge about genetic testing and confidence when discussing *BRCA1/2* testing.

### Inclusion criteria

Participants had to be actively engaged in discussing *BRCA1* and *BRCA2* genetic testing and/or their results in breast cancer or genetics service setting but could be from any appropriate discipline and at any level or seniority. Interested participants had to provide fully informed consent which included an agreement to attend the entire 8-h workshop and to complete all pre and post-workshop assessments.

Workshops were accredited 10 Continuing Professional Development (CPD) points from the Royal College of Physicians. Brighton & Sussex Medical School Research Governance and Ethics Committee approved the study (ref: ERA/RMLS21/6/1) which was funded by the Breast Cancer Research Foundation.

### Assessments

#### Communication

In the week prior to workshop attendance, participants were audio-recorded responding to six different videotaped questions, two from each of the three family members (patient simulators), via Zoom. Questions covered areas including the risks of developing breast or ovarian cancer in TNBC, when and what relatives should be told, implications of HRT and risk if *BRCA1/2* positive (for a pathogenic variant), RRS, screening and demands from an anxious individual for RRS. Question selection for each participant was based on their speciality e.g. surgeon/oncologist, specialist nurse, geneticist, or genetic counsellor. The maximum possible communication score was 28.

#### Self‑confidence questionnaire

A study-specific ten-item questionnaire similar to that used in previous assessments of our educational interventions [7] was adapted for attendees to self-rate their own confidence pre and post-workshop when discussing risk and uncertainties in breast cancer genetics, on a scale from 1 (not at all) to 10 (very confident) (see Supplementary Table S1). They also gave examples of the types of personality/ emotional characteristics of individuals or situations that they found most challenging in the clinic.

#### Knowledge questionnaire

Attendees completed an 18 item Multiple Choice Questionnaire (MCQ) (see Supplementary Table S2) developed by us to assess knowledge about genetic testing in people with and without breast cancer and which would be required to enable appropriate communication with the proband, and other family members depicted in the workshop scenarios. The answers could also be derived from discussions held during the workshop and from the interview with the geneticist (GE).

#### Self‑awareness, tolerance of uncertainty and numeracy skills

Participants completed the 12 item Intolerance of Uncertainty Scale [14] which measures Inhibitory Anxiety (IA) which is uncertainty inhibiting action in ambiguous situations and prospective anxiety (PA)—fear of future events. Individuals with a high intolerance of uncertainty are often risk-averse; patients with a high intolerance can be reluctant to even receive their genetic risk results [15]. HCPs may convey their own high intolerance through nuanced communication with patients. The scale has utility therefore when examining the decision-making of both parties. In one study for example oncologists with a high intolerance of uncertainty were not confident about discussing risk of recurrence and the exclusion of adjuvant chemotherapy with patients when disclosing gene expression profiling scores even when these were low or intermediate [16].

During workshops, attendees completed a short 4-item numeracy exercise based on Schwartz [17] examining their ability to convert probabilities into proportions, proportions into percentages and vice versa, important skills to enable better patient understanding of risk.

At the workshop conclusion, before participants left the venue, the knowledge MCQ, communication and self-confidence assessments were conducted again. Attendees also rated the quality of the educational materials, specific aspects of the content, and whether or not they would recommend attendance to colleagues.

#### Hypotheses

Our a priori hypotheses were that post-workshop (1) HCPs knowledge about genetic testing, including the communication skills required for effective consultations would improve; (2) HCPs would feel more confident when discussing *BRCA1/2* testing and its implications with patients and relevant family members.

#### Statistical analyses

For the rating of communication, an independent data manager assigned random numbers to each audio-recorded interview. Researchers (VJ & LF), each blinded to time-point, used a study-specific coding checklist, to rate independently key points of knowledge or appropriate reassurance in response to each answer. The rate-rerate reliability agreement of researchers’ own coding scores of participants’ recordings and inter-rater reliability checks on 10% of each other’s ratings were performed using intraclass correlations.

The total scores for communications skills (from the audio recordings), knowledge (MCQ), HCP’s intolerance of uncertainty and self-reported confidence levels, are all described using means, standard deviations (SDs), medians, and interquartile ranges (IQRs) by participants’ speciality and overall.

As most participants showed improved scores post workshop across all the measurements, we estimated mean post-course changes (i.e., post-minus precourse participant’s scores), their 95% confidence intervals (CIs) and tested the null hypothesis of zero mean change, assuming a t-distribution for the scores changes to quantify these improvements. Each item analysed reflects a distinctive communication area, the interpretation of which is of interest on its own. We do not aim to make an overall communication recommendation based on the amalgamation of all items; therefore, corrections for multiple testing are not necessary. The results are exploratory, and all inferential statements are to be interpreted individually for each item [18, 19].

We also fitted linear regression analyses to HCP’s self-confidence levels on each area of information post-course with intolerance of uncertainty scale as an explanatory variable. Diagnostic plots, including plots of residuals, and Q–Q plots were used to check the model assumptions. The goodness of fit was assessed through the model’s R-squared statistic. The analyses were carried out using the statistical software R [20].

## Results

Between June and October 2021, 7 facilitator-led face-to-face workshops were attended by 53 HCPs (21 surgeons 5 oncologists, 18 specialist breast care/consultant nurses and 9 genetic counsellors). All were experienced senior HCPs apart from three genetic counsellors, one surgeon and one oncologist and one nurse, who were either more junior or trainees. Attendees had all participated in various unspecified previous training in genetics generally, but none apart from the genetic counsellors, reported having had any specific communication skills training in genetics.

### Basic numeracy

Table 1 shows the percentage of participants' correct responses to the four basic numeracy items. The poorest performances were for questions 2, which required conversion of a proportion to a percentage and 3, which required calculation of the expected frequency of an event based on its probability of occurrence, and which only 36/53 (68%) and 35/53 (66%) respectively answered correctly.

**Table 1:** Basic numeracy.

	Question	*n*/53	%
Q1	A person taking Drug A has a 1% chance of an allergic reaction. If 1000 people take the drug, how many will have a reaction?	51	96.2
Q2	A person taking Drug B has a 1 in a 1000 chance of an allergic reaction. What percentage of people taking the drug will have a reaction?	36	67.9
Q3	The chances of getting a serious viral infection is 0.0005. How many of 10,000 exposed people might get the infection?	35	66.0
Q4	Imagine I flip a fair coin 1000 times. How many times will the coin land heads up?	47	88.7

### Self-confidence

#### Primary problems areas identified by participants pre-workshop

The primary personality type that participants felt less confident dealing with were individuals with an anxious predisposition (21/53; 40%). Other issues found challenging included: those wanting RRS but who are not eligible on the NHS, discussions with people who have a strong family history but who prove negative (for a pathogenic variant) when tested, and those who do not wish to disclose these positive results to the family, those with indeterminate findings and advising individuals who are pathogenic gene variant carriers about HRT (see also further examples in Supplementary Table S3).

**Fig. 2:**
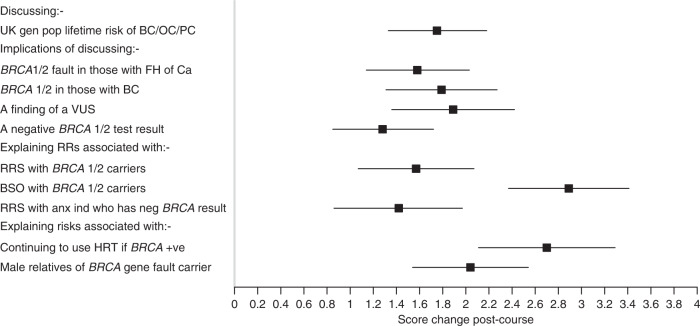
Forest plot of responses to the self- confidence questionnaire.

Figure 2 (Forest plot) shows that participants’ self-confidence when discussing various issues concerned with genetic testing and treatment implications all improved significantly post workshop (see also Supplementary Tables S1 and S4). Improvements were seen irrespective of discipline.

#### Intolerance of Uncertainty Scale (IUS)

IUS scores by speciality are shown in Table 2. As a group, participants had scores showing a high tolerance for uncertainty. Low self-confidence when discussing variants of uncertain significance was associated with higher intolerance to uncertainty scores (beta = −0.09, 95% CI: −0.16 to −0.02; *P* = 0.012, *R*-squared = 0.12).

**Table 2:** Intolerance of uncertainty scale (IUS).

	Surgeons/oncologists (*n* = 26)			Genetic counsellors (*n* = 9)			Nurses (*n* = 18)			Overall (*n* = 53)		
	Total	PA	IA	Total	PA	IA	Total	PA	IA	Total	PA	IA
Mean	26.92	18.65	8.27	28.22	18.11	10.11	26.83	17.83	9	27.11	18.28	8.83
SD	6.42	5.27	1.66	7.9	4.83	3.69	7.59	4.9	3.16	6.96	4.99	2.67
Median	25.5	17.5	8	28	19	10	26	17	8.5	27	18	8
IQR	21.25–31	14–23	7–10	22–36	14–22	8–12	23–29.5	15–19	7–10.75	22–31	14–22	7–10
Range	17–39	9–28	6–16	17–37	11–25	6–14	15–47	10–29	5–18	15–47	9–29	5–18

*PA*   prospective anxiety, *IA*   inhibitory anxiety.

#### Knowledge scores

Table 3 shows participants’ total knowledge scores by speciality and overall. Total knowledge scores improved for all participants irrespective of speciality, the mean change post workshop was 7.06 (95% CI: 6.37–7.74; *P* < 0.001). There were more correct answers post workshop to all questions except for Q4 ‘what proportion of all triple-negative BC are due to a *BRCA1* or *BRCA2* fault (pathogenic variant)’ (see Supplementary Table S5).

**Table 3:** Mean, standard deviation (SD), median and interquartile range (IQR) of HCPs knowledge total score (scale 1–18) pre and post workshop.

	Surgeons/oncologists (*n* = 26)		Genetic counsellors (*n* = 9)		Nurses (*n* = 18)		Overall (*n* = 53)		
	Before	After	Before	After	Before	After	Before	After	Score change
Mean	7.58	15.19	10.22	15.67	7.44	14.5	7.98	15.04	7.06
SD	2.06	1.9	2.77	0.87	2.33	1.98	2.46	1.82	2.48
Median	8	16	11	16	7	14.5	8	16	7
IQR	6–9	14–16.75	8–13	15–16	6–9	13–16	6–9	14–16	6–8

#### Communication

The inter-rater agreement between two coders was good (ICC = 0.63, 95% CI: 0.12–0.89), and rate-rerate consistency was high (ICC = 0.75, 95% CI: 0.17–0.94; ICC = 0.97, 95% CI: 0.85–0.99, respectively). The wide 95% CI for the inter-rater agreement is due to the small sample size.

A majority of participants’ answers to questions posed by the proband and her family members were rated significantly higher post workshop compared to that pre-workshop (47/53 (88.7%) were higher, 4 participants (one surgeon, 2 nurses, and 1 genetic counsellor) worsened slightly (1–2 points) and score of 2 stayed the same). Table 4 shows the communication scores by speciality. The overall mean change post workshop was 5.38 (95% CI: 4.37–6.38; *P* < 0.001).

**Table 4:** Mean, standard deviation (SD), median and interquartile range (IQR) of HCPs’ recorded answers to proband and family members’ questions total score (scale 0–28) pre and post workshop.

	Surgeons/oncologists (*n* = 26)		Genetic counsellors (*n* = 9)		Nurses (*n* = 18)		Overall (*n* = 53)		
	Before	After	Before	After	Before	After	Before	After	Score change
Mean	9.88	15.42	11.67	17.44	9.89	14.83	10.19	15.57	5.38
SD	3.5	3.6	3.24	3.68	2.4	3	3.14	3.47	3.64
Median	10.5	15.5	12	16	10.5	15	11	15	6
IQR	7.25–12.75	13–18.75	10–14	15–20	8–11.75	12.25–17	8–12	13–18	3–8

#### Opinion of workshop

Feedback at the end of the workshop revealed that all attendees regarded it as useful, informative, and enjoyable. All (100%) would ‘definitely’ recommend the programme to their colleagues.

## Discussion

The primary objective hypotheses of the TRUSTING educational programme evaluation were that post-workshop HCPs’ knowledge base about genetic testing, and the communication skills required for effective consultations, would measurably improve.

The subjective hypothesis was that HCPs would also feel more confident about discussing *BRCA1/2* testing and its implications with patients and relevant family members.

All hypotheses, both objective and subjective were clearly demonstrated permitting confidence in the educational value of the programme.

Mainstream genetic testing pathways may prove to be an important means of enabling the identification of breast cancer patients carrying germline pathogenic variants earlier access to (1) more appropriate targeted treatments; (2) risk-reducing procedures; and (3) counselling and testing of relevant family members. The success of such programmes does depend on good multidisciplinary team working and effective communication regarding the implications of genetic testing. There is disquiet however amongst some clinicians regarding their own ability to discuss genetic testing and the resulting management options for patients and affected family members regarding further risk-reducing and prevention strategies [4]. A recent systematic review showed that HCPs often displayed limited understanding of general genetic concepts and had low confidence about integrating these routinely into clinical care [3]. Another recent scoping review identified the range of both genetic and genomic learning needs of oncologists and oncology nurses and highlighting that tailored support, education and training was required to improve their confidence and skills [21].

The literacy and numeracy of most general populations is low thus HCPs need to be adept at describing complex information in flexible ways without being patronising. In England, 43% of adults do not have adequate skills to understand health information only 61% have adequate numeracy skills to understand risk [22, 23]. The statistical literacy and numeracy of many HCPs is also worrying, as their advice may influence patients’ decisions. One study showed that HCPs could not draw mathematically correct inferences from probabilistic screening information regarding results following screening for Down Syndrome, yet the 2/3rd of obstetricians who gave incorrect answers were confident they were correct [24]. Some TRUSTING workshop participants felt embarrassed by revelations of their own poor numeracy and welcomed the opportunity to consider easy ways to explain numbers, percentages, probabilities and risk.

Educational programmes designed to improve communication may fail to change behaviours effectively if the methods used or the contents of them are inappropriate. Our systematic review conducted prior to the development of TRUSTING revealed that there were few publications evaluating interventions explicitly designed to help HCPs discussing hereditary breast cancer risk and testing and that most failed to operationalise the skills that were included, outcome measures or analysis [12]. More recently, an influential report on the guidelines for genomics education has been published which might assist future programme developers [25]. Communication interventions that have been shown to change skills that transfer into a clinic setting and which are enduring need three essential components: elements that expand or solidify participants’ knowledge base, communication skills development and personal awareness [7, 26].

We incorporated all these essentials into TRUSTING.

## Limitations

Due to the COVID pandemic restrictions, we were unable to conduct the communication assessments with patient simulators pre and post workshop face to face so used videotaped recordings of the proband and family members asking questions. Attendees’ responses were audio-taped which some found challenging. Despite the potential lack of realism this involved, significant improvements in communication were nevertheless observed post workshop. Attendees had varied experience and training in genetics, all self-selected to participate and were motivated to engage actively with the workshops. It will be interesting to see if the observed benefits are achieved with a wider roll-out of the educational programme. We also acknowledge that the primary focus of the workshops was centred around one fictitious family with a *BRCA2* pathogenic variant and that further evidence is needed demonstrating that a similar programme with different variants and scenarios would necessarily be equally effective.

## Conclusions

If mainstreaming of genetic testing is to deliver benefits for patients, families and HCPs, then more attention needs to be paid to training. Evaluation of the TRUSTING programme showed that it significantly improved the knowledge, communication skills and self-confidence of attendees sufficiently for us to consider offering the programme further to more clinical cancer teams. We intend to use the same methodology to develop other scenarios for prostate and colorectal cancer.

## Supplementary information

Supplementary Tables 1-5

reproducibility checklist

## Data Availability

The data that support the findings of this study are available upon request from the corresponding author.
