# Supplementary material for: Talking about Risk, UncertaintieS of Testing IN Genetics (TRUSTING): development and evaluation of an educational programme for healthcare professionals about BRCA1 & BRCA2 testing
Source: Br J Cancer. 2022 Jun 17;127(6):1116–22. doi: 10.1038/s41416-022-01871-x (PMC9470577; doi:10.1038/s41416-022-01871-x)
Supplement: Supplementary file 1 — Supplementary Tables 1-5 [file 41416_2022_1871_MOESM1_ESM.docx]

**Supplementary Tables S1 – S5**

**Table S1: Self-confidence Questions and Percentage (number correct /n) of correct answers pre and post workshop**

|  | **Table S1. Mean (SD) of HCPs self-reported confidence levels (scale 1-10) pre and post workshop by speciality and overall score change** | | | | | | | | |  |
| --- | --- | --- | --- | --- | --- | --- | --- | --- | --- | --- |
|  |  | **Surgeons/oncologists (n=26)** | | **Genetic Counsellors (n=9)** | | **Nurses (n=18)** | | **Overall (n= 53)** | | |
|  | How confident are you discussing… | Before | After | Before | After | Before | After | Before | After | Score change |
| 1 | UK general population lifetime risks | 6.08 (1.9) | 8.23 (0.86) | 7.22 (1.72) | 8.33 (1.12) | 5.78 (2.02) | 7.28 (1.64) | 6.17 (1.94) | 7.92 (1.28) | 1.75 (1.53) |
| 2 | *BRCA*1 or 2 with individual with FH of cancer | 6.31 (2.15) | 8.35 (0.69) | 8 (1.32) | 8.78 (1.09) | 6.61 (1.94) | 7.94 (1) | 6.7 (2.02) | 8.28 (0.91) | 1.58 (1.62) |
| 3 | *BRCA*1 or 2 with individual who have had breast cancer | 6.23 (2.21) | 8.58 (0.81) | 7.78 (1.39) | 8.56 (1.01) | 6.72 (2.16) | 8.22 (1.06) | 6.66 (2.12) | 8.45 (0.93) | 1.79 (1.74) |
| 4 | Variant of uncertain significance | 5.15 (2.48) | 7.04 (1.8) | 7.11 (1.54) | 8.56 (1.51) | 4.83 (2.66) | 6.94 (1.83) | 5.38 (2.51) | 7.26 (1.83) | 1.89 (1.92) |
| 5 | Negative *BRCA*1 or 2 test result | 6.62 (1.79) | 8.12 (0.99) | 7.89 (1.36) | 8.67 (1.22) | 6.56 (2.06) | 7.78 (1.22) | 6.81 (1.86) | 8.09 (1.13) | 1.28 (1.57) |
| 6 | Risk reductions associated with bilateral mastectomy | 6.69 (2.56) | 8.12 (1.61) | 7.33 (1.22) | 8.22 (0.83) | 6.17 (2.26) | 8.28 (1.18) | 6.62 (2.28) | 8.19 (1.35) | 1.57 (1.81) |
| 7 | Risk reductions associated with bilateral salpingo-oophorectomy | 4.81 (2.02) | 8.04 (0.92) | 7.11 (1.17) | 8.67 (0.5) | 4.56 (2.33) | 7.61 (1.09) | 5.11 (2.19) | 8 (0.98) | 2.89 (1.89) |
| 8 | Risk Reducing Surgery with anxious individual | 6 (2.12) | 7.77 (1.07) | 6.78 (1.2) | 7.22 (1.09) | 6.06 (2.24) | 7.44 (0.86) | 6.15 (2.02) | 7.57 (1.01) | 1.42 (2) |
| 9 | The risks of continuing to use HRT | 5.19 (2.35) | 7.92 (0.84) | 4.67 (2.06) | 7.78 (1.09) | 5.22 (2.05) | 7.67 (0.84) | 5.11 (2.17) | 7.81 (0.88) | 2.7 (2.14) |
| 10 | Testing male relatives of a *BRCA* gene fault carrier | 5.92 (1.85) | 8.46 (0.76) | 8.22 (1.2) | 9.11 (0.93) | 6.22 (2.13) | 8.11 (1.32) | 6.42 (2.01) | 8.45 (1.05) | 2.04 (1.81) |

**Table S2: TRUSTING KNOWLEDGE Multiple Choice Questionnaire**

**General Questions**

1. What proportion of the UK general population carry a BRCA 1 or BRCA 2 fault?
2. 1:800
3. 1:300
4. 1:80
5. don’t know
6. What proportion of all UK breast cancer cases is caused by an inherited BRCA 1 or BRCA 2 fault?
7. ~2%
8. ~5%
9. ~10%
10. don’t know
11. What proportion of the Ashkenazi Jewish population have a fault in BRCA 1 or BRCA 2 genes?
12. 1:10
13. 1:25
14. 1:40
15. don’t know
16. What proportion of all triple negative BC are due to a BRCA1 or BRCA 2 fault?
17. ~5%
18. ~10%
19. ~30%
20. don’t know
21. Which gene fault has a higher lifetime risk of ovarian cancer?
22. BRCA 1
23. BRCA 2
24. both the same risk
25. don’t know
26. Ovarian cancer in women over 60 years with no family history or personal history of breast cancer is more likely to be BRCA 2 than BRCA 1
27. true
28. false
29. don’t know
30. At what age do UK guidelines suggest MRI breast screening be considered for high risk women?
31. <25 years
32. 30 years
33. >40 years
34. don’t know
35. According to UK guidelines what does the 10 year risk of developing BC in young women have to be before annual MRI screening can start?
36. 5%
37. 8%
38. 10%
39. don’t know
40. What are the lifetime risks of developing BC for a post-menopausal woman with a BRCA 2 gene fault and a family history of BC in her?
41. 60-70%
42. 45-55%
43. 30-40%
44. don’t know
45. What does SNP stand for?
46. single nuclear peptide
47. single nucleotide polymorphism
48. standard nuclear peptide
49. don’t know
50. What is the UK lifetime risk of prostate cancer for men with a BRCA 2 gene fault?
51. ~3-6%
52. ~12-15%
53. ~25-30%
54. don’t know
55. How much does 10 years of combined HRT, increase the risk of BC in women who are BRCA 2 positive?
    1. decreases the risk
    2. zero effect
    3. doubles the risk
    4. don’t know
56. Which one of these in general is most likely to increase BC risk in women?
    1. never being pregnant
    2. having first child after the age of 36
    3. having more than 4 children
    4. don’t know

**Looking at the family tree**

**Anna aged 49, had WLE for TNBC, is having chemotherapy and recently tested positive for BRCA 2**

1. Would you advise her to have risk reducing bilateral mastectomy
2. immediately after her chemotherapy finishes
3. whenever she feels ready
4. never, not necessary
5. don’t know

**Josephine aged 54, is BRCA2 positive, nulliparous and taking combined HRT**

1. Which surgical risk-reducing management strategy would you suggest Josephine prioritises?
2. bilateral mastectomy
3. bilateral salpingo-oopherectomy
4. both
5. don’t know
6. Josephine has been taking combined HRT for 3 years, would your initial advice be to:-
7. stop taking it now
8. continue with oestrogen only HRT
9. not worry at the moment
10. don’t know

**Emma aged 35, has tested negative for BRCA2 but is deeply anxious that she will develop breast cancer**

1. Despite the negative BRCA result, is Emma’s lifetime risk of developing BC
2. same as the general population
3. moderately higher than the general population
4. much higher than the general population
5. don’t know
6. At what age can she start having mammographic screening?
7. as from now
8. 50 years
9. 40 years
10. don’t know

**Table S3: Typical challenging personality characteristics & situations cited during workshops**

| Those so anxious they cannot alter their perceptions of risk even when the facts are explained |
| --- |
|  |
| Individuals who have pre-conceived ideas regarding their personal risk of developing breast cancer and where their expectation is that they must have a gene test. |
| Those who already have an agenda possibly based on inaccurate information |
|  |
| Super anxious people and patients who want exact answers for clinical situation with variable outcomes |
|  |
| Very anxious individuals who have multiple sources of information. Often there are several consultations and often the same questions are discussed several times |
|  |
| Anxious information-seekers where further information is increasing anxiety. |
|  |
| Very chatty anxious patients |
|  |
| Patients anxious to proceed with risk reducing surgery immediately following the finding of a BRCA mutation especially if < 30 years |
|  |
| Extremely anxious people who are not really listening to what you are saying. |
|  |
| Patients who find it difficult to take in the information (which is complex!) |
|  |
| Patients who are very fixated on mastectomies when their personal risks are not of a magnitude to justify it |
|  |
| Women who want to have risk reducing surgery who have tested BRCA negative but still have significant family history and are therefore afraid they are at very high risk |
|  |
| You see different family members on separate occasions and the history is conflicting |
|  |
| Where a patient with a known genetic problem does not wish to inform her relatives |
|  |
| Patients who pay thousands of pounds on genomic/pharmacological reports on their cancer where most of the outputs have not actionable mutations |
|  |
| When Genetic results are negative but there is clearly a strong family history |
|  |
| Where someone is adamant they would like testing but are not eligible on the NHS as they do not meet the testing criteria. Have to discuss private testing. |
|  |
| Generally where my knowledge is limited, or at the edge of my expertise, much like much of the inherited breast cancer scenarios are |
|  |
| Testing very young people where no current actionable intervention |

**Table S4. Post-workshop score change (95% confidence intervals) of HCPs self-reported confidence levels**

| How confident are you discussing … | **Score change** | **95% CI** | **P-value** |
| --- | --- | --- | --- |
| UK general population lifetime risks | 1.75 | 1.33 to 2.18 | <0.001 |
| *BRCA*1 or 2 with individual with FH of cancer | 1.58 | 1.14 to 2.03 | <0.001 |
| *BRCA*1 or 2 with individual who have had breast cancer | 1.79 | 1.31 to 2.27 | <0.001 |
| Variant of uncertain significance | 1.89 | 1.36 to 2.42 | <0.001 |
| Negative *BRCA* 1 or 2 test result | 1.28 | 0.85 to 1.72 | <0.001 |
| Risk reductions associated with bilateral mastectomy | 1.57 | 1.07 to 2.07 | <0.001 |
| Risk reductions associated with bilateral salpingo-oophorectomy | 2.89 | 2.37 to 3.41 | <0.001 |
| Risk Reducing Surgery with anxious individual | 1.42 | 0.86 to 1.97 | <0.001 |
| The risks of continuing to use HRT | 2.7 | 2.11 to 3.29 | <0.001 |
| Testing male relatives of a *BRCA* gene fault carrier | 2.04 | 1.54 to 2.54 | <0.001 |

**Table S5: Knowledge Scores from MCQ (SF2), Percentage (number correct /n) of correct answers pre and post workshop**

|  | **Surgeons/oncologists (n=26)** | | **Genetic Counsellors (n=9)** | | **Nurses (n=18)** | | **Overall (n=53)** | |
| --- | --- | --- | --- | --- | --- | --- | --- | --- |
| **Question** | **Before** | **After** | **Before** | **After** | **Before** | **After** | **Before** | **After** |
| 1 | 31% (8/26) | 96% (25/26) | 67% (6/9) | 89% (8/9) | 11% (2/18) | 89% (16/18) | 30% (16/53) | 92% (49/53) |
| 2 | 23% (6/26) | 92% (24/26) | 11% (1/9) | 56% (5/9) | 22% (4/18) | 83% (15/18) | 21% (11/53) | 83% (44/53) |
| 3 | 38% (10/26) | 96% (25/26) | 56% (5/9) | 100% (9/9) | 44% (8/18) | 72% (13/18) | 43% (23/53) | 89% (47/53) |
| 4 | 35% (9/26) | 31% (8/26) | 67% (6/9) | 33% (3/9) | 56% (10/18) | 44% (8/18) | 47% (25/53) | 36% (19/53) |
| 5 | 54% (14/26) | 69% (18/26) | 78% (7/9) | 100% (9/9) | 39% (7/18) | 39% (7/18) | 53% (28/53) | 64% (34/53) |
| 6 | 46% (12/26) | 88% (23/26) | 22% (2/9) | 78% (7/9) | 56% (10/18) | 100% (18/18) | 45% (24/53) | 91% (48/53) |
| 7 | 88% (23/26) | 92% (24/26) | 100% (9/9) | 100% (9/9) | 78% (14/18) | 94% (17/18) | 87% (46/53) | 94% (50/53) |
| 8 | 31% (8/26) | 69% (18/26) | 78% (7/9) | 100% (9/9) | 22% (4/18) | 56% (10/18) | 36% (19/53) | 70% (37/53) |
| 9 | 27% (7/26) | 92% (24/26) | 44% (4/9) | 56% (5/9) | 44% (8/18) | 89% (16/18) | 36% (19/53) | 85% (45/53) |
| 10 | 81% (21/26) | 96% (25/26) | 89% (8/9) | 100% (9/9) | 39% (7/18) | 83% (15/18) | 68% (36/53) | 92% (49/53) |
| 11 | 31% (8/26) | 88% (23/26) | 89% (8/9) | 100% (9/9) | 44% (8/18) | 94% (17/18) | 45% (24/53) | 92% (49/53) |
| 12 | 54% (14/26) | 96% (25/26) | 67% (6/9) | 100% (9/9) | 56% (10/18) | 100% (18/18) | 57% (30/53) | 98% (52/53) |
| 13 | 12% (3/26) | 88% (23/26) | 11% (1/9) | 100% (9/9) | 22% (4/18) | 89% (16/18) | 15% (8/53) | 91% (48/53) |
| 14 | 62% (16/26) | 92% (24/26) | 100% (9/9) | 100% (9/9) | 89% (16/18) | 94% (17/18) | 77% (41/53) | 94% (50/53) |
| 15 | 27% (7/26) | 88% (23/26) | 44% (4/9) | 100% (9/9) | 28% (5/18) | 89% (16/18) | 30% (16/53) | 91% (48/53) |
| 16 | 4% (1/26) | 50% (13/26) | 33% (3/9) | 56% (5/9) | 11% (2/18) | 33% (6/18) | 11% (6/53) | 45% (24/53) |
| 17 | 46% (12/26) | 92% (24/26) | 44% (4/9) | 100% (9/9) | 33% (6/18) | 100% (18/18) | 42% (22/53) | 96% (51/53) |
| 18 | 69% (18/26) | 100% (26/26) | 22% (2/9) | 100% (9/9) | 50% (9/18) | 100% (18/18) | 55% (29/53) | 100% (53/53) |
